# Rv0579 Is Involved in the Resistance to the TP053 Antitubercular Prodrug

**DOI:** 10.3389/fmicb.2020.00292

**Published:** 2020-02-25

**Authors:** Giorgia Mori, Beatrice Silvia Orena, Laurent R. Chiarelli, Giulia Degiacomi, Olga Riabova, José Camilla Sammartino, Vadim Makarov, Giovanna Riccardi, Maria Rosalia Pasca

**Affiliations:** ^1^Department of Biology and Biotechnology “Lazzaro Spallanzani,” University of Pavia, Pavia, Italy; ^2^Bach Institute of Biochemistry, Federal Research Centre “Fundamentals of Biotechnology” of the Russian Academy of Sciences, Moscow, Russia

**Keywords:** Rv0579, drug resistance, prodrug, tuberculosis, antitubercular drug

## Abstract

Tuberculosis remains one of the leading causes of death from a single pathogen globally. It is estimated that 1/4 of the world’s population harbors latent tuberculosis, but only a 5–10% of patients will develop active disease. During latent infection, *Mycobacterium tuberculosis* can persist unaffected by drugs for years in a non-replicating state with low metabolic activity. The rate of the successful tuberculosis treatment is curbed by the presence of these non-replicating bacilli that can resuscitate after decades and also by the spread of *M. tuberculosis* drug-resistant strains. International agencies, including the World Health Organization, urge the international community to combat this global health emergency. The thienopyrimidine TP053 is a promising new antitubercular lead compound highly active against both replicating and non-replicating *M. tuberculosis* cells, with an *in vitro* MIC of 0.125 μg/ml. TP053 is a prodrug activated by the reduced form of the mycothiol-dependent reductase Mrx2, encoded by *Rv2466c* gene. After its activation, TP053 releases nitric oxide and a highly reactive metabolite, explaining its activity also against *M. tuberculosis* non-replicating cells. In this work, a new mechanism of TP053 resistance was discovered. *M. tuberculosis* spontaneous mutants resistant to TP053 were isolated harboring the mutation L240V in Rv0579, a protein with unknown function, but without mutation in *Rv2466c* gene. Recombineering method demonstrated that this mutation is linked to TP053 resistance. To better characterize Rv0579, the protein was recombinantly produced in *Escherichia coli* and a direct interaction between the Mrx2 activated TP053 and Rv0579 was shown by an innovative target-fishing experiment based on click chemistry. Thanks to achieved results, a possible contribution of Rv0579 in *M. tuberculosis* RNA metabolism was hypothesized, linked to toxin anti-toxin system. Overall, these data confirm the role of Rv0579 in TP053 resistance and consequently in the metabolism of this prodrug.

## Introduction

Tuberculosis (TB), an old disease considered tied to the past, started again capturing the attention for different reasons, such as the spread of *Mycobacterium tuberculosis* drug-resistant strains (Koch et al., 2018). In the world, in 2018 about 500,000 people were infected by *M. tuberculosis* strains resistant to the most effective first-line drug rifampicin (RR-TB), and 78% of these isolates were multidrug-resistant (MDR-TB). Three countries accounted for almost half of the world’s cases of MDR-TB: India, China and the Russian Federation (World Health Organization [WHO], 2019). In order to fight drug-resistant TB, the treatment options have become limited, thus stressing the need for new therapies.

Another important threat is latent TB (LTB); according to WHO, LTB corresponds to a persistent immune response to stimulation by *M. tuberculosis* antigens without evidence of active TB (World Health Organization [WHO], 2018). The possible progression of LTB to active disease is a complex condition determined by bacterial, host, and environmental factors (Getahun et al., 2015).

About one-quarter of the world population develops LTB and it is considered as a persistent reservoir of active infection. Active TB will develop in 5–15% of individuals with LTB during their lifetimes. Several comorbidities could be related with increased risk of developing active TB, such as the HIV co-infection (Furin et al., 2019; Huaman and Sterling, 2019). Consequently, an effective treatment for LTB should be mandatory.

Current recommended LTB treatments include one of the following options: once-weekly isoniazid plus rifapentine for 3 months, daily rifampin for 4 months, daily isoniazid plus rifampin for 3–4 months, and daily isoniazid for 6–9 months (Huaman and Sterling, 2019). Treatments based on rifamycins seem to cause less adverse events and, consequently, to improve the therapy completion (Villa et al., 2019).

New and more effective drugs against LTB with fewer adverse events are needed in order to prevent active TB and to shorten treatment duration.

In this context, the thienopyrimidine (TP) TP053 is a new promising prodrug killing both replicating (MIC = 0.125 μg/ml) and non-replicating (MIC = 0.8 μg/ml) *M. tuberculosis* cells (Albesa-Jové et al., 2014). By microbiological, genetic, biochemical and crystallographic studies, its mechanism of activation was elucidated; TP053 is a prodrug activated by the reduced form of the DsbA-like mycoredoxin Mrx2 (encoded by *Rv2466c* gene), a mycothiol-dependent reductase with an unusual active-site motif CXXC (Albesa-Jové et al., 2014; Rosado et al., 2017). Mrx2 utilizes a chaperone-like mechanism of conformational changes, mainly in the CXXC active site motif, to recognize TP053 and promoting compound reduction (Albesa-Jové et al., 2015).

Recently, it has been demonstrated that Mrx2 works as a nitroreductase causing a release of nitric oxide (NO) from TP053 (Chiarelli et al., 2020). Thus, conceivably TP053 globally affects *M. tuberculosis* cell growth by NO release, which could be the cause of its activity against non-replicating bacilli, in a similar way to the NO releasing drug pretomanid, upon Ddn activation (Singh et al., 2008).

The study of the *M. tuberculosis* metabolic pathways affected by TP053 exposure, through transcriptional analysis, revealed features consistent with NO release, confirming the characteristic mechanism of action of TP053 against non-replicating bacilli (Chiarelli et al., 2020). Furthermore, it was identified a highly reactive metabolite, 2-(4-mercapto-6-(methylamino)-2-phenylpyrimidin-5-yl)ethan-1-ol, produced upon Mrx2 transformation of TP053, which could be co-responsible for the antimycobacterial effects on replicating and non-replicating *M. tuberculosis* cells (Chiarelli et al., 2020). However, no potential target of this active metabolite has been identified so far.

In this work, we demonstrated that the Rv0579 protein has a role in the mechanism of resistance of TP053, even if its physiological role in *M. tuberculosis* still remains unclear.

## Materials and Methods

### Bacterial Strains and Growth Conditions

Cloning steps were performed in *Escherichia coli* XL1-Blue, following standard methods (Sambrook and Russell, 2001) and using the oligonucleotides described in Supplementary Table S1. Protein expression was achieved in *E. coli* BL21(DE3).

*Mycobacterium tuberculosis* H37Rv and mutant strains were grown aerobically at 37°C either in Middlebrook 7H9 broth (Difco) or on Middlebrook 7H11 agar (Difco), both supplemented with 10% OADC Middlebrook Enrichment. When necessary, antibiotics were added at the following concentrations: ampicillin 100 μg/ml, hygromycin 200 μg/ml (20 μg/ml for *M. tuberculosis*), and kanamycin 50 μg/ml (20 μg/ml for *M. tuberculosis*).

All the experiments with *M. tuberculosis* were performed in Biosafety level 3 laboratory by authorized and trained researchers.

### MIC Determination

MICs for the compounds were determined by means of the micro-broth dilution method. Dilutions of *M. tuberculosis* wild-type and mutant cultures (about 10^5^–10^6^ CFU/ml) were streaked onto 7H11 solid medium containing a range of drug concentrations. Plates were incubated at 37°C for about 21 days and the growth was visually evaluated. The lowest drug dilution at which visible growth failed to occur was taken as the MIC value. Results were expressed as the average of at least three independent replicates.

### Isolation and Characterization of *M. tuberculosis* Mutants Resistant to TP053

The isolation of *M. tuberculosis* mutants was performed by plating ∼10^10^ cells from an exponential growth phase culture of *M. tuberculosis* H37Rv onto 7H11 medium containing different concentrations of TP053, ranging from 5- to 40-fold the MIC of the wild-type strain. Genomic DNA of *M. tuberculosis* resistant mutants was isolated and sequenced by using Illumina HiSeq2000 technology at IGA Technology Services S.R.L. (Udine, Italy). For the bioinformatic analysis of Illumina data, repetitive PE and PPE gene families were discarded as well as SNPs and Indels with less than 50% probability. The mutations identified were confirmed by Sanger sequencing (Eurofins Genomics), after PCR amplification using the primers described in Supplementary Table S1. PCR products were purified using the Wizard^®^ SV Gel and PCR Clean-Up system (Promega).

### Recombineering Method

Recombineering method utilizing mycobacteriophage-encoded functions was used in order to confirm the role of *Rv0579* mutation in TP053 resistance, as previously described (van Kessel and Hatfull, 2008). Briefly, a *M. tuberculosis* strain that carries the recombineering plasmid pJV62 was induced by acetamide in mid-logarithmic growth and electrocompetent cells were prepared. Electroporation was performed with different concentrations (100 and 500 ng) of Rv0579rec oligonucleotide (Supplementary Table S1), which anneals to the template for discontinuous DNA synthesis (lagging strand), thus increasing recombination frequency. The introduced point mutation was co-selected by plating the transformations onto a 7H11 plate containing TP053 (1.25 and 5 μg/ml corresponding to 10× and 40× MIC, respectively) and kanamycin (20 μg/ml).

### Expression and Purification of *M. tuberculosis* Rv0579

*Mycobacterium tuberculosis* H37Rv *Rv0579* gene from genomic DNA was amplified by standard PCR, using the primers reported in Supplementary Table S1, and PCR fragments were cloned in the pET-SUMO vector (Invitrogen), to give pET-SUMO/*Rv0579* recombinant plasmid. Rv0579 enzyme was produced in *E. coli* BL21(DE3), by overnight induction with 0.5 mM isopropyl-β-thiogalactopyranoside (IPTG) at 25°C. Thereafter, cells were harvested, re-suspended in 150 ml of buffer A (50 mM Tris–HCl pH 7.5, 300 mM NaCl, 0.5 mM dithiothreitol (DTT), 5% glycerol, 2 mM MgCl_2_) containing 1 mM PMSF, disrupted by sonication and centrifuged for 30 min at 50,000 × *g*. The supernatant was loaded on HisTrap Crude column (1 ml, GE Healthcare), washed with 50 ml of buffer A containing 50 mM of imidazole, then Rv0579 elution was performed with 250 mM of imidazole in buffer A. The eluted protein was dialyzed using a HiPreP Desalting 26/10 column equilibrated in 50 mM Tris–HCl pH 7.5, 50 mM imidazole, 100 mM NaCl, 0.5 mM DTT, 2 MgCl_2_, 5% glycerol, incubated at 4°C in the presence of 15 μl SUMO-protease and then re-purified on HisTrap Crude column. The purified protein was checked by SDS-PAGE, concentrated to 5 mg/ml, and stored at −80°C. The protein concentration was determined by absorbance at 280 nm (ε: 21,680 M^–1^ cm^–1^).

### Target Fishing

To demonstrate a direct interaction between Rv0579 protein and TP053, a TP053-azide-PEG3-biotin conjugate complex was used.

The 11526119 derivative of TP053, prepared as described in Supplementary Material, was firstly characterized to verify that it showed the same features as TP053. For this reason, Mrx2 enzymatic activity using 11526119 as substrate was assayed as previously described (Albesa-Jové et al., 2014), to confirm that this compound is activated like TP053.

Then, 11526119 was used to perform azide-alkyne cycloaddiction reaction with Azide-PEG3-biotin conjugate, to form an adduct which can be immobilized on a streptavidin agarose resin (Supplementary Figure S1). To this purpose, in a final volume of 500 μl, 11526119 (200 μM) was incubated with 2-fold excess of Azide-PEG3-biotin conjugate, in the presence of 250 μM of CuSO_4_ and 5 mM Na-ascorbate, overnight at room temperature. The reaction was checked by thin layer chromatography, then incubated 1 h with Streptavidine Agarose resin, and washed with phosphate saline buffer (PBS) to remove any unbound material.

The complex was incubated with Rv0579 (0.5 mg/ml) in a final volume of 200 μl, in the presence of Mrx2 (2 mg/ml, prepared according to Albesa-Jové et al., 2014), DTT (1 mM) and *Mycobacterium smegmatis* methanolic extract (2%) at 37°C for 30 min, then incubated 1 h with Streptavidine Agarose resin. After the incubation, the resin was washed five times with PBS to remove unbound proteins and then analyzed in SDS-PAGE. As negative control, the same incubation was performed with the only streptavidine-agarose resin, or in absence of *M. smegmatis* methanolic extract, to avoid Mrx2 activation.

### Quantitative Real-Time PCR

Quantitative Real-Time PCR was performed on RNA extracted from *M. tuberculosis* H37Rv and *Rv0579* mutant strains, both treated with TP053 (0.06 μg/ml; 0.5× MIC) or untreated using the Direct-zol^TM^ RNA MiniPrep Kit (Zymo Research). The cDNA was obtained from about 1 μg of total RNA by the Quantitec reverse transcription kit (Qiagen). The RT-PCR experiments were performed using the QuantiTect SYBR Green PCR Master Mix kit (Qiagen) and the “Rotor Gene 6000” thermocycler (Corbett Life Science).

The reaction was carried out in a final volume of 15 μl and contained: 7.5 μl of 2× SYBR Green Buffer (supplied by the kit), cDNA, primers (7.5 pmol) and RNase-free water. All reactions were repeated in triplicate and the mean value was considered. The primers used to assess the transcriptional analysis of *sigA* and *Rv0579* genes are present in Supplementary Table S1.

## Results

### The Mutation in *Rv0579* Gene Is Responsible for TP053 Resistance

To identify the TP053 cellular target(s), two *M. tuberculosis* mutants resistant to TP053 (MIC = 2.5 μg/ml, 20× MIC of wild type strain) have been isolated using a *M. tuberculosis* culture overexpressing *mrx2* gene, coding for the activator (*M. tuberculosis*/pSODIT-*mrx2*) (Albesa-Jové et al., 2014). In this way, the selection of mutation(s) in *mrx2* could be avoided. After the confirmation of the TP053 resistance, to find the mutation(s) responsible for TP053 resistance, whole genome sequencing (WGS) of the new isolated resistant mutants was performed using Illumina method (Table 1). Interestingly, *M. tuberculosis* 2466.3 and 2466.4 mutants revealed the same mutation in *Rv0579* gene (C718G → L240V), coding for a conserved hypothetical protein (Table 1). The presence of the mutation in the *Rv0579* gene was confirmed by Sanger sequencing. No mutation was detected in *mrx2* gene on either the chromosome or the plasmid.

**TABLE 1:** Characteristics of the *M. tuberculosis* spontaneous TP053 resistant mutants.

***M. tuberculosis* strains**	**Concentration of isolation of TP053 resistant colonies (μg/ml)**	**Mutation in *mrx2***		**Mutation in *Rv0579* (aa change)**	**MIC to TP53 (μg/ml)**
		**On genome**	**On plasmid**		
*M. tuberculosis*/pSODIT/*mrx2*	–	–	–	–	0.125
2466.3 mutant	0.5 (4× MIC)	No	No	C718G (L240V)	2.5 (20× MIC)
2466.4 mutant	1 (8× MIC)	No	No	C718G (L240V)	2.5 (20× MIC)

The specific contribution of the mutation in *Rv0579* to TP053 resistance was confirmed by recombineering method (van Kessel and Hatfull, 2008) using different TP053 concentrations (10× and 40× MIC of the wild type strain), thus allowing the generation of the C718G substitution in chromosomal *Rv0579* of wild-type H37Rv (Supplementary Table S2). The generated TP053 resistant colonies were screened for the introduction of the *Rv0579* C718G substitution by Sanger sequencing and their TP053 resistance profile confirmed by MIC determination. None of the recombineering-generated mutants harboured mutations in *mrx2*. In every tested mutant, the only presence of *Rv0579* mutation (C718G) conferred TP053 resistance (MIC = 2.5 μg/ml, 20× MIC of wild type strain). These results confirmed the role of Rv0579 in the mechanism of resistance to TP053.

Thus, the possibility that the resistance to TP053 might be due to the overexpression of *Rv0579* gene was excluded by quantitative PCR of *Rv0579* transcripts from both *M. tuberculosis* wild-type and TP053 resistant (Rec5 mutant obtained by recombineering) strains, in presence/absence of prodrug; in fact, no significant difference in the expression of *Rv0579* was observed (Supplementary Table S3).

### Rv0579 Protein Is Able to Directly Interact With the Activated TP053

In order to further investigate the role of Rv0579 in the TP053 resistance, the Rv0579 protein was recombinantly produced in *E. coli* and purified to homogeneity (Supplementary Figure S2). The produced protein was found to be bound to nucleic acids. Subsequent DNase and RNase digestion revealed RNA to be the main contaminant (Supplementary Figure S3). However, an efficient digestion of RNA was achieved only after partial denaturation of the protein, indicating a strong interaction. For this reason, DNase and RNase were added in the lysis buffer (100 μl, 2 mg/ml) for additional purification steps. Despite several attempts using different chromatographical approaches, the nucleic acids were not completely removed in any case (data not shown).

**FIGURE 1:**
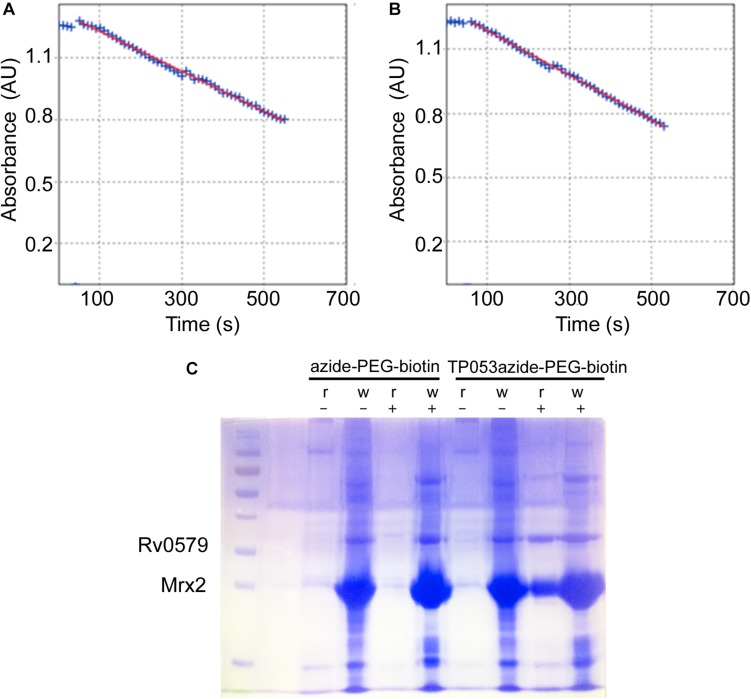
Target fishing experiments. **(A,B)** The enzymatic activity assay of Mrx2 using 11526119 as a substrate **(A)**, was compared with the assay using TP053 **(B)** demonstrating that the compound retaining the same capability to be metabolized by the enzyme. Then, it was suitable for target fishing experiments. **(C)** SDS-PAGE analysis of the target fishing experiments. The streptavidine-agarose resin was incubated 1 h with the TP53-azide-PEG-biotin, or with azide-PEG-biotin complex, reacted with Rv0579. After the incubation the samples were washed with buffer, and both resin and wash fractions analyzed by SDS-PAGE. r, resin; w, wash; +, incubation with Rv0579 in the presence of mycothiols, to allow Mrx2 activation; –, incubation with Rv0579 without mycothiols as blank control. Rv0579 did not show any reactivity with the streptavidine-agarose resin alone, since the protein was found only in wash fractions (lanes 5 and 6). By contrast, Rv0579 was found bound the resin-TP053 complex, but only after Mrx2 activation (lane 9). This experiment demonstrates a direct interaction between the protein and the activated compound, which confirms a role of Rv0579 in resistance to TP053.

The achieved recombinant protein was then exploited to demonstrate a possible direct interaction between Rv0579 and the activated TP053 through a target-fishing experiment based on click chemistry (Figure 1). For this experiment, a TP053 derivative (11526119; MIC = 0.5 μg/ml) (Supplementary Material) was used, which was shown to retain TP053 ability to be activated by Mrx2 by an *in vitro* enzymatic assay (Figures 1A,B). Consequently, the Rv0579 protein was incubated with a TP053azide-PEG3-biotin conjugate complex in presence of the activator Mrx2, then bound on Streptavidin Agarose resin. After several washing steps, the resin was analyzed by SDS-PAGE (Figure 1C). As control, the same reaction was performed also in the absence of mycothiols, to avoid Mrx2 activation, or using the only azide-PEG3-biotin.

The obtained data demonstrated that the activated prodrug binds not only to Mrx2, as expected, but also to Rv0579 (Figure 1C), indicating a direct interaction between the activated TP053 and Rv0579.

These results confirm the role of Rv0579 in the mechanism of TP053 resistance.

### Understanding the Role of Rv0579 in TP053 Resistance

Previous RNA-seq experiments demonstrated that the exposure to TP053 (6 μg/ml; ∼50× MIC) of *M. tuberculosis* cells caused an over-expression of some toxin-antitoxin (TA) systems (e.g.: HigAB, VapBC16) (Chiarelli et al., 2020).

Interestingly, on *M. tuberculosis* genome, *Rv0579* is located near to *Rv0581* (coding for the antitoxin VapB26) and *Rv0582* (coding for the toxin VapC26) (Kang et al., 2017). Taking advantage of the availability of our *Rv0579* mutant (Rec5 mutant obtained by recombineering), we performed Real-Time PCR using both *M. tuberculosis* wild type and *Rv0579* mutant cultures treated with TP053 (0.06 μg/ml; 0.5× MIC) (Supplementary Table S3) to investigate possible changes in the expression levels of the genes encoding VapBC26 TA system. Untreated cultures were used as control.

Surprisingly, only in *M. tuberculosis* wild type strain an up-regulation of the gene encoding the VapC26 toxin was found; in the *Rv0579* mutant the Rv0582 over-expression was not detected (Supplementary Table S3). These results suggest a possible link between Rv0579 and VapBC26 system.

Since Rv0579 protein possesses the two domains Ub-Mut7C and Mut7-C, usually related to RNase activity (Marchler-Bauer et al., 2017), we hypothesised that the link with VapBC26 could involve this possible activity. Unfortunately, we were not able to determine it, because of the nucleic acid contamination of the purified protein (data not shown). Further experiments to support this hypothesis currently were limited until this issue is solved.

## Discussion

TP053 is a very promising compound active against replicating and non-replicating *M. tuberculosis* cells with a peculiar mechanism of action (Albesa-Jové et al., 2014; Chiarelli et al., 2020). In fact, this prodrug is activated by Mrx2 producing an active metabolite by NO release. Furthermore, TP053 inhibits *M. tuberculosis* cell growth by influencing several metabolic pathways such as RNA metabolism (Chiarelli et al., 2020).

In this work, a new mechanism of resistance to TP053 was discovered. *M. tuberculosis* spontaneous mutants resistant to TP053 were isolated harboring a mutation in *Rv0579* gene. The contribution of this mutation to TP053 resistance was confirmed by recombineering method. Moreover, target fishing experiment demonstrated that Rv0579 is able to bind TP053, but only upon Mrx2 activation of the compound.

*Rv0579* is a non-essential gene (Griffin et al., 2011), encoding a conserved hypothetical protein with unknown function. The sequence of Rv0579 protein shows two important domains, usually linked to RNase activity: Ub-Mut7C and Mut7-C (Marchler-Bauer et al., 2017) (Supplementary Table S4).

Mut7-C is a RNAse domain of the PIN fold with an inserted zinc ribbon located at the C terminus of the protein (Anantharaman et al., 2002); this domain is typical of VapC toxin (toxin-antitoxin system). The mutation found in TP053 resistant *M. tuberculosis* mutants is located at the end of this domain. Ub-Mut7C domain is occasionally present at the N-terminus of the protein with the Mut7-C domain, suggesting an RNA-binding role (Iyer et al., 2006) (Supplementary Table S4). In our case, this hypothesis is also corroborated by the finding that the recombinant protein tightly binds RNA (Supplementary Figure S3).

Iyer et al. (2006) identified a group of proteins containing the two domains, Ub-Mut7C and Mut7-C, fused; Rv0579 belongs to this group containing several proteins with unknown function belonging to different bacterial species (Supplementary Figure S4 and Supplementary Table S4). Interestingly, the alignment of these proteins shows that the mutation in Rv0579 responsible for TP053 resistance is present at the end of Mut7-C domain and it corresponds to a change of an amino acid conserved in some bacterial species (L240; *Streptomyces coelicolor*, *Nostoc punctiforme*, *Nocardia farcinica*, *Thermobifida fusca*) (Supplementary Figure S4). In other tested species, this amino acid changes in Met.

It is noteworthy that TP053 releases NO, consequently affecting RNA metabolism and causing inhibition of respiration, oxidative stress, and DNA damage (Chiarelli et al., 2020). This again suggests that Rv0579 has a role in RNA metabolism, one of the pathways mainly affected by NO release (Darwin et al., 2003; Voskuil et al., 2003; Manjunatha et al., 2009).

From the achieved results, and thanks to a deep analysis of the Rv0579 sequence, a role of Rv0579 in RNA metabolism could be hypothesized, linked to toxin anti-toxin systems (e.g. VapBC26). Indeed, TP053 treatment in wild-type *M. tuberculosis* caused an up-regulation of the *Rv0582* gene encoding the VapC26 toxin, while no effect has been seen in *M. tuberculosis* resistant mutant carrying mutation in *Rv0579*. These evidences thus suggest a role of this protein in the regulation of this toxin-antitoxin system.

Even if the physiological role of Rv0579 is still under investigation, this work unveils some important aspects of the protein, paving the way for further evidences that will allow to clearly elucidate its role in *M. tuberculosis* metabolism and in TP053 resistance.

## Data Availability Statement

All datasets generated for this study are included in the article/Supplementary Material.

## Author Contributions

GM, BO, LC, and MP designed the study. LC, VM, GR, and MP wrote the manuscript and interpreted the data. GM, BO, LC, GD, OR, and JS performed the experiments. All authors approved the final version of the manuscript.

## Conflict of Interest

The authors declare that the research was conducted in the absence of any commercial or financial relationships that could be construed as a potential conflict of interest.
