# Supplementary material for: Rv0579 Is Involved in the Resistance to the TP053 Antitubercular Prodrug
Source: Front Microbiol. 2020 Feb 25;11:292. doi: 10.3389/fmicb.2020.00292 (PMC7052010; doi:10.3389/fmicb.2020.00292)
Supplement: Supplementary file 1 [file Data_Sheet_1.pdf]

Supplementary Material

1 Supplementary Data

Synthesis of 11526119

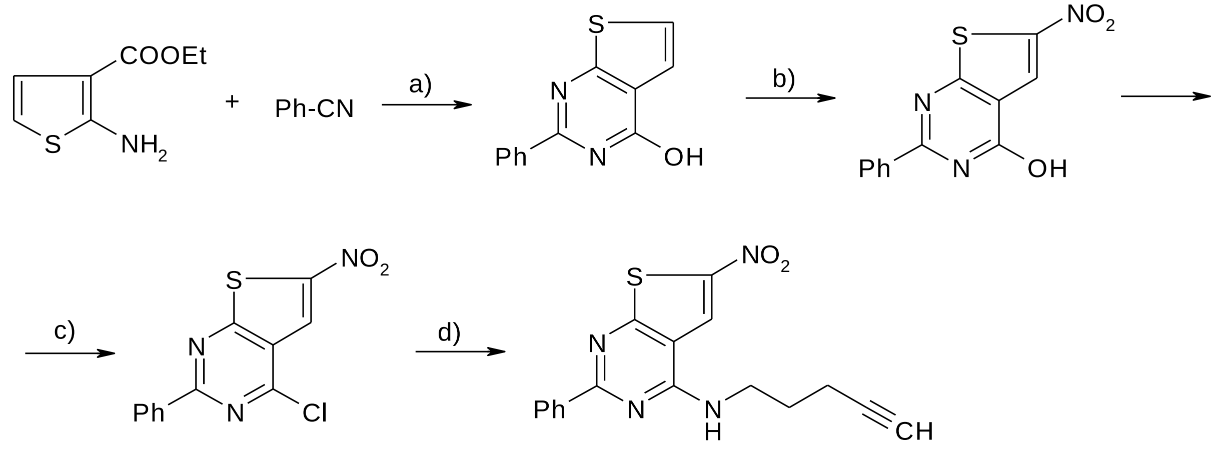

a) A mixture of ethyl 2-aminothiophene-3-carboxylate (4 g, 23.4 mmol) and benzonitryl (2.5 mL, 24.4 mmol) in dioxane solution of hydrochloric acid (~21 %, 55 ml) in glass bomb was mixed at 110ºC for 6 h. The heating was discontinued and reaction masse was mixed additional 16 h at 20ºC. Formed solid was filtered off, washed by dioxane, water and methanol. 2-Phenylthieno[2,3-d]pyrimidin-4-ol was used in the next step without additional purification. Yield is 30%. Mp. 238-40ºC. Mass (EI), *m/z* (I_{relat}.(%)): 229.2708 $[M]^+$ (76). $C_{12}H_8N_2OS$. ^1H NMR (DMSO-d6), δ: 12.70 (br s, 1H, OH), 8.15 (m, 2H, 2CH), 7.55 (m, 4H, 4CH), 7.42 (d, 1H, *J* = 6.0 Hz, CH) ppm.

b) Solid 2-phenylthieno[2,3-d]pyrimidin-4-ol (1.14 g, 5 mmol) was solved in conc. sulfuric acid (15 ml), stored for 40 min at 20°C, cooled to -10ºC and treated by small portions by solid potassium nitrate (0.6 g, 6 mmol), with temperature inside reaction not more than -5ºC. The reaction masse was hold at this temperature additional 30 min and mixed with ice. Formed solid of 6-nitro-2-phenylthieno[2,3-d]pyrimidin-4-ol was washed by water till pH ~7 and used in the next step without additional purification. Yield is 89%. Mp. 321-3 ºC. Mass (EI), *m/z* (I_{relat}.(%)): 274.2684 $[M]^+$ (45). $C_{12}H_7N_3O_3S$. ^1H NMR (DMSO-d6), δ: 13.20 (br s, 1H, OH), 8.30 (s, 1H. CH), 8.17 (d, 2H, 2 CH), 7.60 (m, 3H, 3 CH) ppm.

c) A mixture of 6-nitrothieno[2,3-d]pyrimidine-4-ol (1.80 g, 6.54 mmol), phosphorus oxichloride (18 eq.) and trienthylamine hydrochlorided (0.83 g, 6.0 mmol) was refluxed for 1 h. The reaction masse was cooled, poured onto ice, formed solid was filtered off. The yield of 4-chloro-6-nitro-2-phenylthieno[2,3-d]pyrimidine is 99%. Mp. 210-3ºC. Mass (EI), *m/z* (I_{relat}.(%)): 292.7138 $[M]^+$ (62). $C_{12}H_6ClN_3O_2S$. ^1H NMR (CDCl3), δ: 8.55 (m, 2H, 2CH), 8.26 (s, 1H, CH), 7.58 (m, 3H, 3CH) ppm.

d) Solution of 4-chloro-6-nitro-2-phenylthieno[2,3-d]pyrimidine (0.1 g, 0.34 mmol) in dioxane (5 ml) was treated by solution of pent-4-yn-1-amine (0.056 g, 0.64 mmol) in MeOH (1.5 ml) at 20°C. After 30 min the reaction mixture was evaporated on vacuum and residue was treated by water. Solid precipitate was filtered off and washed by water and methanol. The yield of 6-nitro-N-pent-4-yn-1-yl-2-phenylthieno[2,3-d]pyrimidin-4-amine is 64%. Mp. 232-4ºC (EtOAc). Mass (EI), *m/z* (I_{relat}.(%)): 339.3848 $[M]^+$ (76). $C_{17}H_{14}N_4O_2S$. ^1H NMR (DMSO-d6), δ: 8.80 (br s, 1H, NH), 8.30 (br s, 1H, CH), 8.40 (m, 2H, 2CH), 7.50 (br s, 3H, 3CH), 3.75 (br d, 2H, CH_2), 2.75 (br s, 1H, CH), 2.30 (br s, 2H, CH_2), 1.85 (m, 2H, CH_2) ppm.

2 Supplementary Figures and Tables

TABLE S1 | Oligonucleotides used in this study.

Oligonucleotide	Sequence 5'-3'	Purpose
**sigAF**	GATGACGACGAGGAGAT	Real-Time PCR of *sigA*
**sigAR**	GCCGATCTGTTTGAGGTA	
**0579rtF**	GACGTCAACCTCGGCCAG	Real-Time PCR of *Rv0579*
**0579rtR**	GAATTCGCTGCTCGCCCA	
**Rv0579rec**	AGTTAGCTGGTCCCGCAGTCGTTCGACGA<br>CGCGAACCAACCGTGCGTGGTGAGATCCCGG	Insertion of c718g mutation by recombineering
**0579SumoFor**	ATGGTCGGCTATGTGGA	Cloning of Rv0579 in pET-SUMO vector
**0579SumoRev**	TCAGGTCGAAGTAGTTAG	
**Rv2466cseqFor**	GGAACAGGTGCGGGGCGG	Sequencing of *mrx2* (*Rv2466c)*
**Rv2466cseqRev**	TGCCCGACATTGTGCCCG	
**Rv0579seqFor**	TGCTTCGGCTGTTGGGCT	Sequencing of *Rv0579*
**Rv0579seqRev**	CATTGGTGGCGGGGACA	

TABLE S2 | MIC to TP053 of *M. tuberculosis* recombineering mutants.

**Number of TP053 resistant colonies obtained by recombineering**	**Concentration of isolation (TP053, µg/ml)**	**Mutation observed in *Rv0579* after recombineering (nucleotidic change)**	**MIC to TP053 (µg/ml)**
4	1.25 (10X MIC)	C718G	2.5 (20X MIC)
1	5 (40X MIC)	C718G	2.5 (20X MIC)

TABLE S3 | Expression levels of *Rv0579* in *M. tuberculosis* wild type and mutant strains.

***M. tuberculosis* strains**	***Rv0579***	***Rv0581*** (antitoxin)	***Rv0582*** (toxin)
H37Rv	1.00 ± 0.028	1.00 ± 0.015	1.00 ± 0.01
Rec5	0.93 ± 0.011	**2.71 ± 0.14**	6.02 ± 0.18
H37Rv + TP053 (0.5X MIC)	1.33 ± 0.11	0.50 ± 0.03	**12.31 ± 0.23**
Rec5 + TP053 (0.5X MIC)	0.73 ± 0.17	**2.37 ± 0.08**	2,58 ± 0.057

TABLE S4 | Characteristics of Rv0579 protein (252 aa).

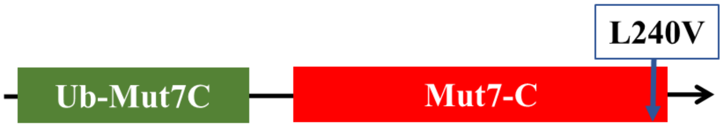

Domain	Description of Family domain	Start	End
Ub-Mut7C	This domain is found at the N-terminus of bacterial proteins with Mut7-C, suggesting an RNA-binding role.	2	82
Mut7-C	RNAse domain of the PIN fold with an inserted Zinc Ribbon at the C terminus.	97	241*

* The mutation is L240V.

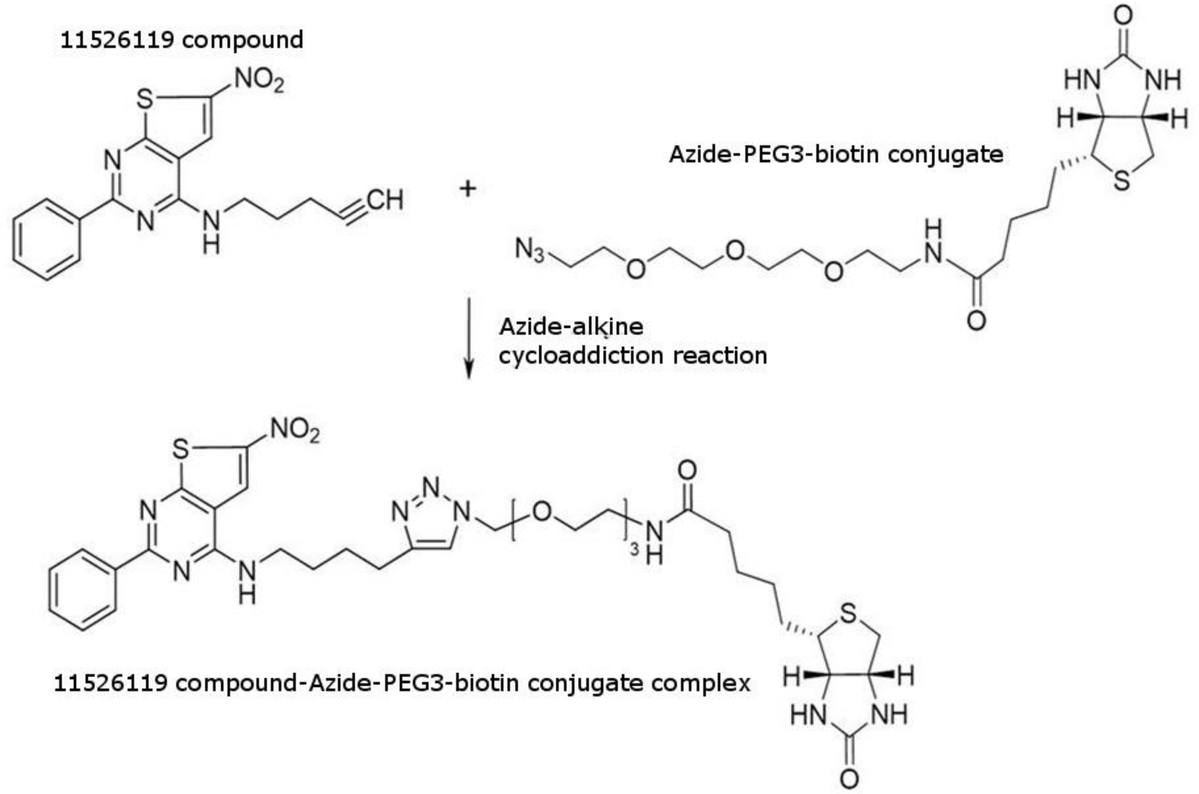

Figure S1. Cycloaddiction reaction of 11526119 compound with Azide-PEG3-biotin conjugate.

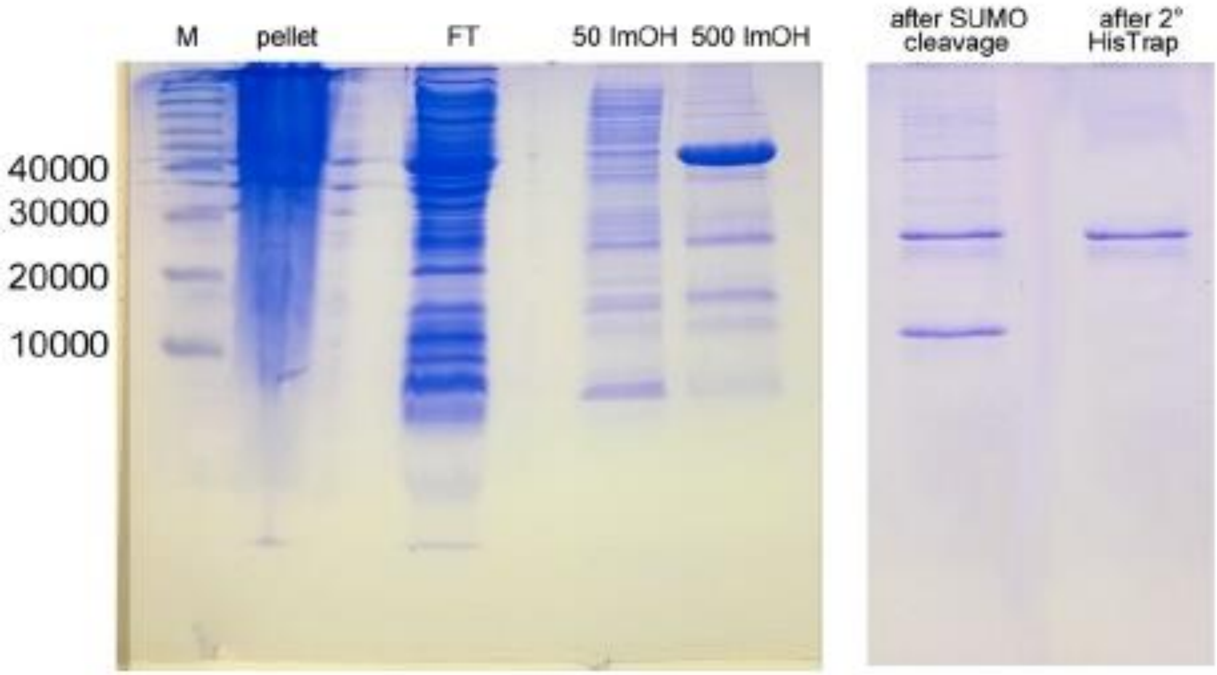

Figure S2. SDS-PAGE of the Rv0579 purification steps.

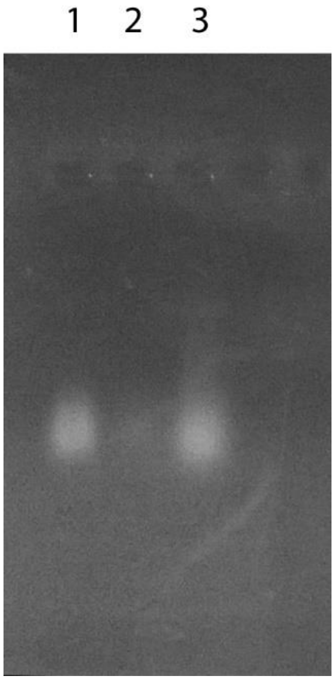

Figure S3. 1% agarose gel electrophoresis of the purified Rv0579 protein, before (lane 1) and after digestion with RNase (lane 2) or DNase (lane 3). Purified Rv0579 was incubated for 5 minutes at 90°C, then after nuclease addition the samples were incubated at 37°C for 10 minutes and analyzed.

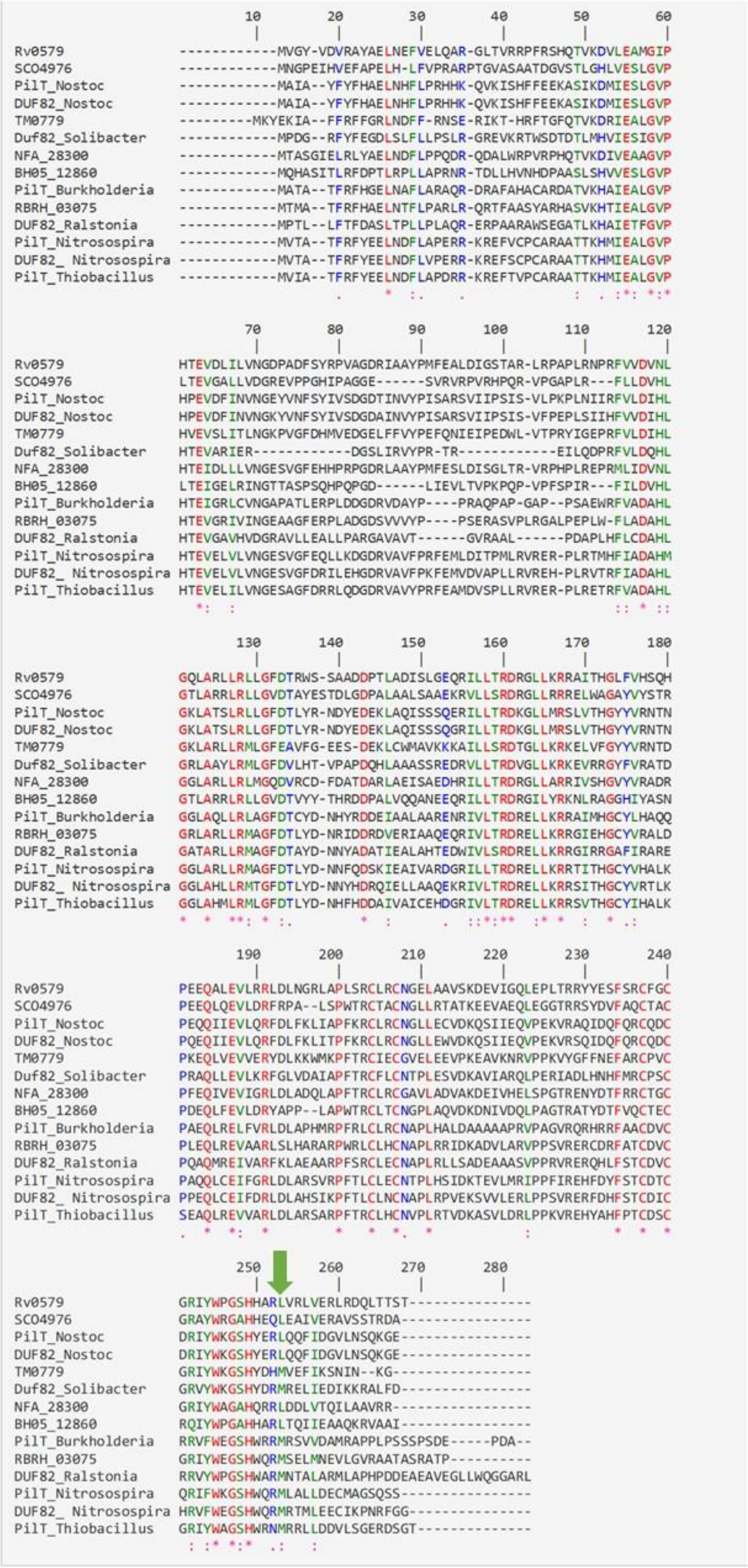
Rv0579
SCO4976
PilT_Nostoc
DUF82_Nostoc
TM0779
Duf82_Solibacter
NFA_28300
BH05_12860
PilT_Burkholderia
RBRH_03075
DUF82_Ralstonia
PilT_Nitrosospira
DUF82_ Nitrosospira
PilT_Thiobacillus

Figure S4. Alignment by ClustalW of Rv0579 with some orthologues bacterial proteins.

In green is indicated the position of mutation in Rv0579. In the alignment there are the following proteins: SCO4976 of *Streptomyces coelicolor* A3(2); PilT_Nostoc of *Nostoc punctiforme* NIES-2108; DUF82_Nostoc of *Nostoc punctiforme*; TM0779 of *Thermotoga maritima* MSB8; DUF82_Solibacter of *Solibacter usitatus*; NFA_28300 of *Nocardia farcinica* IFM 10152; BH05_12860 of *Thermobifida fusca*; PilT-Burkholderia of *Burkholderia cenocepacia*; RBRH_03075 of *Paraburkholderia rhizoxinica* HKI 454; DUF82_Ralstonia of *Ralstonia solanacearum* PSI07 (plasmid); PilT_Nitrosospira of *Nitrosospira multiformis*; DUF82_ *Nitrosospira of Nitrosospira multiformis* ATCC 25196; PilT_Thiobacillus of *Thiobacillus denitrificans*.
